# Applicability of human-specific STR systems, GlobalFiler™ PCR Amplification Kit, Investigator 24plex QS Kit, and PowerPlex® Fusion 6C in chimpanzee (*Pan troglodytes*)

**DOI:** 10.1186/s13104-021-05632-6

**Published:** 2021-05-29

**Authors:** Abhishek Singh, Vivek Sahajpal, Mukesh Thakur, Lalit Kumar Sharma, Kailash Chandra, Deepika Bhandari, Arun Sharma

**Affiliations:** 1 Zoological Survey of India, New Alipore, Kolkata, 700 053 West Bengal India; 2 State Forensic Science Laboratory, Directorate of Forensic Services, Junga, Shimla, 171218 Himachal Pradesh India

**Keywords:** GlobalFiler™ PCR amplification kit, Investigator 24plex QS kit, PowerPlex® fusion 6C system, Chimpanzee, Human identification

## Abstract

**Objectives:**

Human identification systems based on STRs are widely used in human population genetics and forensic analysis. This study aimed to validate the cross-reactivity of three widely known human-specific STR identification systems i.e. GlobalFiler™ PCR Amplification Kit, Investigator 24plex QS Kit, and PowerPlex® Fusion 6C in chimpanzee.

**Results:**

The present study revealed the successful amplification of 18 loci using GlobalFiler™ PCR Amplification Kit, 18 loci using Investigator 24plex QS Kit, and 20 loci using PowerPlex® Fusion 6C system. The marker Amelogenin (AMEL) showed differential allele size between male and female revealing the gender identity of chimpanzees and thus validates their application concerning forensic examination, population estimation, and genetic analysis.

**Supplementary Information:**

The online version contains supplementary material available at 10.1186/s13104-021-05632-6.

## Introduction

Human DNA profiling is a well-known method of identification in the field of forensic science. Earlier, the forensic DNA analysis was used to perform in high profile cases or particularly in cases where the other identification methods such as fingerprinting and anthropometric features failed to produce the identity due to the unavailability of required evidence. With technological inventions and scientific developments, DNA profiling became less needed and rather regularised in general practice. In the light of this practice, several profiling kits based on Short Tandem Repeats (STRs), starting from four (Quadruplex) to 27 (PowerPlex Fusion 6C) markers are developed [1]. While developing these marker kits, validations were carried out on several aspects including species specificity [2]. Validation of species specificity is a critical parameter as the forensic casework samples may encounter non-human DNA in an actual or fabricated crime scene and therefore cross-reactivity of markers are critical to assess [3]. The most common non-human DNA which is used for the assessment of cross specificity belongs to chimpanzees, orangutans, bonobos, gorillas, macaque, cat, horse, dog, cow, sheep, goat and pigs [3]. Among all these animals, non-human primates DNA may amplify within the marker range of humans [3]. Earlier, the STRs specific to humans were thoroughly tested in non human primates for population studies [4], phylogenetics [5] and individual identification of both captive [6] and wild animals [7]. Ely et al. [8] also suggested that the STRs specific to humans could be used for the identification of poached chimpanzees or establishing the pedigree relation among the wild individuals. Further, to assess the extent of amplification of human STR markers in non-human primates, few recent studies i.e. Thakur et al. [9] and Singh et al. [10] performed cross-species validation of several commercial forensic STR kits in chimpanzees. These studies also assessed the power of individual identification of forensically important markers in chimpanzees. This provides a scope of future application of these STR kits in case of challenging samples and individual identification. Therefore, it is essential to validate the cross-reactivity of all the marker sets which are commercially utilized in forensic casework.

In this study, we performed the cross-species assessment of three commercially and widely used human identification STR marker kits i.e. GlobalFiler™ PCR Amplification Kit, Investigator 24plex QS Kit, and PowerPlex® Fusion 6C System on Chimpanzees DNA.

## Main text

### Methods

#### Sample collection and DNA extraction

Two male (Chhotu and Mastan) and one female (Buri) chimpanzee hair samples were received from the Alipore Zoological Garden, Kolkata. Genomic DNA extraction was carried out from the hair follicles using the Qiagen DNeasy Blood and Tissue kit (Qiagen, Germany), following the manufacturer's protocol.

#### PCR amplification and Genotyping

Three most widely used 6 dye multiplex Human STR systems i.e. GlobalFiler™ PCR Amplification Kit (Applied Biosystems, USA), Investigator 24plex QS Kit (Qiagen, Germany), and PowerPlex® Fusion 6C System (Promega, USA) were used for the genotyping of the samples. The GlobalFiler™ PCR amplification Kit includes 21 autosomal STR markers i.e. D3S1358, vWA, D16S539, CSF1PO, TPOX, D8S1179, D21S11, D18S51, D2S441, D19S433, TH01, FGA, D22S1045, D5S818, D13S317, D7S820, SE33, D10S1248, D1S1656, D12S391, D2S1338 with amelogenin (AMEL) as a gender determining marker, DYS391 as Y STR marker and one Y-indel polymorphic marker. The Investigator 24plex QS Kit includes 22 CODIS recommended STR markers i.e. TH01, D3S1358, vWA, D21S11, TPOX, DYS391, D1S1656, D12S391, SE33, D10S1248, D22S1045, D19S433, D8S1179, D2S1338, D2S441, D18S51, FGA, D16S539, CSF1PO, D13S317, D5S818, D7S820 including two quality control sensors QS1 and QS2 and a gender determination marker amelogenin. The PowerPlex® Fusion 6C system following the recommendations of both CODIS and ESS consists of 18 expanded CODIS core loci i.e. CSF1PO, FGA, TH01, vWA, D1S1656, D2S1338, D2S441, D3S1358, D5S818, D7S820, D8S1179, D10S1248, D12S391, D13S317, D16S539, D18S51, D19S433, D21S11 and five highly discriminative loci i.e. Penta D, Penta E, D22S1045, TPOX, and SE33. It also includes two gender determining markers that are amelogenin and DYS391 and two rapidly mutating Y-STR markers i.e. DYS570 and DYS576. For each marker system, separate PCR reactions were carried out in a total volume of 25 μl with both positive and negative controls on the GeneAmp PCR system 9700 thermocycler (Applied Biosystems, USA) following the manufacturer's protocols. Fragment analyses of the amplified products were carried on ABI 3130 Genetic analyzer (Applied Biosystems, USA) following the manufacturer’s instructions [11–13].

#### Data analysis

Genotype calling was performed using GeneMapper ID v.3.2 and the scoring and rearrangement of allelic data were performed in Microsoft Excel. To evaluate the efficiency of Human specific STR identification system in chimpanzees, genetic diversity indices such as observed heterozygosity (Ho), expected heterozygosity (He), unbiased heterozygosity (uHe), observed number of alleles (Na), expected number of alleles (Ne), and fixation Index (F) were obtained using GENEALEX v.6.5 [14]. The polymorphism information content (PIC) for each locus was calculated using the software STRAF (STR Analysis for Forensics) [15]. Genealogical analysis to establish the extent of the relationship was determined by the likelihood of relatedness using ML-Relate [16].

### Results

AMEL successfully assigned gender of known chimpanzees in all three PCR identification kits. Out of the total markers tested with all the kits, three markers of GlobalFiler, three markers including QS1 and QS2 of Investigator 24plex and five markers of PowerPlex® Fusion 6C failed to amplify in all the individuals while the rest of the markers were observed polymorphic (Table 1). Genetic diversity indices based on autosomal STRs revealed a lower heterozygosity estimate in GlobalFiler™ in compare to Investigator 24plex QS and PowerPlex® Fusion 6C System (Table 1). The mean Ho i.e. 0.46 ± 0.08 was observed lower than the mean He of 0.51 ± 0.07 with an average uHe of 0.62 ± 0.08 in the GlobalFiler (Additional file 1: Table S1). The other two kits i.e. Investigator 24plex and PowerPlex® Fusion 6C system showed higher Ho i.e. 0.63 ± 0.08 and 0.68 ± 0.07 with respect to He i.e. 0.61 ± 0.06 and 0.58 ± 0.05 (Additional file 1: Tables S2,S3). Except in the case of Globalfiler amplification kit with a fixation index (F) of 0.06 ± 0.11, the other two kits revealed negative value of fixation index i.e. −0.05 ± 0.10 (Investigator 24plex) and −0.19 ± 0.08 (PowerPlex® Fusion 6C) (Additional file 1: Tables S1–S3). The PIC value revealed that one locus D1S1656 with 0.81 PIC (Globalfiler), two locus D1S1656 and D2S441 with 0.81 PIC (Investigator 24plex) and six locus D1S1656, D2S441, D13S317, D2S1338, D19S433 and D22S1045 with 0.74 PIC (PowerPlex® Fusion 6C) were the most polymorphic loci (Additional file 1: Tables S1–S3). Further, the genealogical relationship analysis based on 13 loci in Globalfiler, 16 loci in both Investigator 24plex and PowerPlex® Fusion 6C revealed that the examined individuals were genetically unrelated with Delta Ln(L) of 9999 (Additional file 1: Table S4).

**Table 1:** Comparative assessment of average genetic diversity estimates of chimpanzees based on STR systems of present and previous studies

STR identification Kit	Allele Size (AMEL)		Total alleles (range)	Failed loci	Na (Mean ± SE)	Ne (Mean ± SE)	Ho (Mean ± SE)	He (Mean ± SE)	uHe (Mean ± SE)	F (Mean ± SE)
	Male	Female								
#GlobalFiler™	98, 104	98	55 (6–1)	D5S818, SE33, D12S391	3.06 ± 0.36	2.74 ± 0.34	0.46 ± 0.08	0.51 ± 0.07	0.62 ± 0.08	0.06 ± 0.01
#Investigator 24plex QS	77, 80	77	67 (6–1)	D12S391, FGA, D7S820	3.72 ± 0.36	3.41 ± 0.35	0.63 ± 0.08	0.61 ± 0.06	0.73 ± 0.07	−0.05 ± 0.10
#PowerPlex® Fusion 6C	80, 86	80	69 (5–1)	Penta E, D5S818, SE33,DYS576, DYS570	3.49 ± 0.29	2.95 ± 0.28	0.68 ± 0.07	0.58 ± 0.05	0.76 ± 0.06	−0.19 ± 0.08
*PowerPlex® 21 System	78, 84	78	58 (5–1)	D5S818, D12S391	3.05 ± 0.28	2.58 ± 0.26	0.93 ± 0.03	0.52 ± 0.05	0.63 ± 0.06	−0.89 ± 0.17
*SureID® 21G	112, 117	112	62 (6–1)	D12S391, D21S11	3.26 ± 0.03	2.84 ± 0.31	0.63 ± 0.07	0.57 ± 0.05	0.68 ± 0.06	−0.13 ± 0.09
*SureID® 23comp	113, 108	108	75 (6–2)	D12S391, D8S1132, D2S441	3.75 ± 0.27	3.31 ± 0.26	0.77 ± 0.05	0.66 ± 0.03	0.82 ± 0.03	−0.17 ± 0.09

*Na* Observed number of alleles, *Ne* effective number of alleles, *Ho* observed heterozygosity, *He* expected heterozygosity, *UHe* unbiased expected heterozygosity, *F* fixation index, ^#^Marker systems used in the present study, *Marker system used in the previous studies

### Discussion

This study attempted to cross-validate the amplification efficiency and gender determination power of human-specific STRs, included in the most commonly used human STR identification system i.e. GlobalFiler™ PCR Amplification Kit, Investigator 24plex QS Kit, and PowerPlex® Fusion 6C System. The marker AMEL successfully identified the gender of three known chimpanzees. The genetic diversity analysis and genealogical relationship analysis revealed higher heterozygosity with several alleles and Delta Ln(L) with a value of 9999 indicating that the examined chimpanzees were genetically unrelated. In the previous attempts of amplification of human specific STR markers in chimpanzees, Thakur et al. [9] and Singh et al. [10] revealed that the majority of the loci showed positive amplifications with SureID® 23comp identification system having highest mean heterozygosity (Table 1). Therefore, the study establishes the fact that the human specific identification systems i.e. GlobalFiler™ PCR Amplification Kit, Investigator 24plex QS Kit, and PowerPlex® Fusion 6C System can be used for the individual identification and forensic analysis of chimpanzees.

Three human STR identification systems can be used for the population assessment and genetic identification in non-human primate-like chimpanzees and also can be tested for their applicability on other non-human primates like orangutans, bonobos, gorillas, macaque and langur.

## Limitations

The study validates the applicability of human-specific STR identification systems in chimpanzees; however, small sample size was the limitation of this study. Thus, we propose further cross-validation on other non-human primates with a large sample size.

## Supplementary Information

**Additional file 1 Table S1.** Genetic diversity indices of chimpanzees with GlobalFiler™ PCR Amplification Kit. **Table S2.** Genetic diversity indices of chimpanzees with Investigator 24plex QS Kit. **Table S3.** Genetic diversity indices of chimpanzees with PowerPlex® Fusion 6C System. **Table S4.** Genealogical relationship of chimpanzees with GlobalFiler™ PCR Amplification Kit, Investigator 24plex QS Kit and PowerPlex® Fusion 6C System.

## Data Availability

All the relevant data is provided as Additional file 1. The raw datasets used and/or analyzed during the current study are available from the corresponding author on reasonable request.
