# Supplementary material for: Applicability of human-specific STR systems, GlobalFiler™ PCR Amplification Kit, Investigator 24plex QS Kit, and PowerPlex® Fusion 6C in chimpanzee (Pan troglodytes)
Source: BMC Res Notes. 2021 May 29;14:212. doi: 10.1186/s13104-021-05632-6 (PMC8164790; doi:10.1186/s13104-021-05632-6)
Supplement: Supplementary file 1 — Additional file 1 Table S1. Genetic diversity indices of chimpanzees with GlobalFiler™ PCR Amplification Kit. Table S2. Genetic diversity indices of chimpanzees with Investigator 24plex QS Kit. Table S3. Genetic diversity indices of chimpanzees with PowerPlex® Fusion 6C System. Table S4. Genealogical relationship of chimpanzees with GlobalFiler™ PCR Amplification Kit, Investigator 24plex QS Kit and PowerPlex® Fusion 6C System. [file 13104_2021_5632_MOESM1_ESM.doc]

Table S1. Genetic diversity indices of chimpanzees with GlobalFiler™ PCR Amplification Kit

Na: Observed number of alleles, Ne: effective number of alleles, Ho: observed heterozygosity, He: expected heterozygosity, UHe: unbiased expected heterozygosity, F: fixation index, PIC: Polymorphism information content, * Loci used in establishing genealogical relationship

| **Locus** | **N** | **Na** | **Ne** | **Ho** | **He** | **uHe** | **F** | **PIC** |
| --- | --- | --- | --- | --- | --- | --- | --- | --- |
| ***D3S1358** | 3 | 4.000 | 3.600 | 0.667 | 0.722 | 0.867 | 0.077 | 0.67 |
| ***vWA** | 3 | 3.000 | 3.000 | 0.000 | 0.667 | 0.800 | 1.000 | 0.59 |
| ***D16S539** | 3 | 4.000 | 3.600 | 0.333 | 0.722 | 0.867 | 0.538 | 0.67 |
| ***CSF1PO** | 3 | 2.000 | 1.800 | 0.667 | 0.444 | 0.533 | -0.500 | 0.35 |
| ***TPOX** | 3 | 2.000 | 1.385 | 0.333 | 0.278 | 0.333 | -0.200 | 0.24 |
| ***D8S1179** | 3 | 5.000 | 4.500 | 0.667 | 0.778 | 0.933 | 0.143 | 0.74 |
| **D21S11** | 1 | 1.000 | 1.000 | 0.000 | 0.000 | 0.000 | #N/A | 0.00 |
| **D18S51** | 2 | 2.000 | 1.600 | 0.500 | 0.375 | 0.500 | -0.333 | 0.30 |
| ***D2S441** | 3 | 5.000 | 4.500 | 0.667 | 0.778 | 0.933 | 0.143 | 0.74 |
| **D19S433** | 2 | 2.000 | 2.000 | 0.000 | 0.500 | 0.667 | 1.000 | 0.38 |
| ***TH01** | 3 | 1.000 | 1.000 | 0.000 | 0.000 | 0.000 | #N/A | 0.00 |
| ***FGA** | 3 | 4.000 | 3.000 | 0.667 | 0.667 | 0.800 | 0.000 | 0.62 |
| **D22S1045** | 2 | 2.000 | 1.600 | 0.500 | 0.375 | 0.500 | -0.333 | 0.30 |
| ***D13S317** | 3 | 3.000 | 2.571 | 1.000 | 0.611 | 0.733 | -0.636 | 0.54 |
| **D7S820** | 2 | 1.000 | 1.000 | 0.000 | 0.000 | 0.000 | #N/A | 0.00 |
| ***D10S1248** | 3 | 4.000 | 3.600 | 0.667 | 0.722 | 0.867 | 0.077 | 0.67 |
| ***D1S1656** | 3 | 6.000 | 6.000 | 1.000 | 0.833 | 1.000 | -0.200 | 0.81 |
| ***D2S1338** | 3 | 4.000 | 3.600 | 0.667 | 0.722 | 0.867 | 0.077 | 0.67 |
| **Mean (± SE)** | 2.67±0.14 | 3.06±0.36 | 2.74±0.34 | 0.46±0.08 | 0.51±0.07 | 0.62±0.08 | 0.06±0.01 |  |

Table S2. Genetic diversity indices of chimpanzees with Investigator 24plex QS Kit

| **Locus** | **N** | **Na** | **Ne** | **Ho** | **He** | **uHe** | **F** | **PIC** |
| --- | --- | --- | --- | --- | --- | --- | --- | --- |
| ***TH01** | 3 | 2.000 | 1.385 | 0.333 | 0.278 | 0.333 | -0.200 | 0.24 |
| ***D3S1358** | 3 | 3.000 | 2.571 | 1.000 | 0.611 | 0.733 | -0.636 | 0.54 |
| ***vWA** | 3 | 3.000 | 3.000 | 0.000 | 0.667 | 0.800 | 1.000 | 0.59 |
| ***D21S11** | 3 | 5.000 | 4.500 | 0.667 | 0.778 | 0.933 | 0.143 | 0.74 |
| ***TPOX** | 3 | 3.000 | 2.571 | 1.000 | 0.611 | 0.733 | -0.636 | 0.54 |
| ***D1S1656** | 3 | 6.000 | 6.000 | 1.000 | 0.833 | 1.000 | -0.200 | 0.81 |
| **SE33** | 1 | 1.000 | 1.000 | 0.000 | 0.000 | 0.000 | #N/A | 0.00 |
| ***D10S1248** | 3 | 5.000 | 4.500 | 0.667 | 0.778 | 0.933 | 0.143 | 0.74 |
| ***D22S1045** | 3 | 4.000 | 3.600 | 0.667 | 0.722 | 0.867 | 0.077 | 0.67 |
| ***D19S433** | 3 | 5.000 | 4.500 | 0.667 | 0.778 | 0.933 | 0.143 | 0.74 |
| ***D8S1179** | 3 | 4.000 | 3.600 | 0.667 | 0.722 | 0.867 | 0.077 | 0.67 |
| ***D2S1338** | 3 | 5.000 | 4.500 | 1.000 | 0.778 | 0.933 | -0.286 | 0.74 |
| ***D2S441** | 3 | 6.000 | 6.000 | 1.000 | 0.833 | 1.000 | -0.200 | 0.81 |
| ***D18S51** | 3 | 4.000 | 3.600 | 0.333 | 0.722 | 0.867 | 0.538 | 0.67 |
| ***D16S539** | 3 | 4.000 | 3.600 | 0.667 | 0.722 | 0.867 | 0.077 | 0.67 |
| ***CSF1PO** | 3 | 2.000 | 1.800 | 0.667 | 0.444 | 0.533 | -0.500 | 0.35 |
| ***D13S317** | 3 | 4.000 | 3.600 | 1.000 | 0.722 | 0.867 | -0.385 | 0.67 |
| **D5S818** | 2 | 1.000 | 1.000 | 0.000 | 0.000 | 0.000 | #N/A | 0.00 |
| **Mean (± SE)** | 2.83±0.12 | 3.72±0.36 | 3.41±0.35 | 0.63±0.08 | 0.61±0.06 | 0.73±0.07 | -0.05±0.10 |  |

Na: Observed number of alleles, Ne: effective number of alleles, Ho: observed heterozygosity, He: expected heterozygosity, UHe: unbiased expected heterozygosity, F: fixation index, PIC: Polymorphism information content, * Loci used in establishing genealogical relationship

Table S3. Genetic diversity indices of chimpanzees with PowerPlex® Fusion 6C System

| **Locus** | **N** | **Na** | **Ne** | **Ho** | **He** | **uHe** | **F** | **PIC** |
| --- | --- | --- | --- | --- | --- | --- | --- | --- |
| **D3S1358** | 2 | 3.000 | 2.667 | 1.000 | 0.625 | 0.833 | -0.600 | 0.55 |
| ***D1S1656** | 3 | 5.000 | 4.500 | 0.667 | 0.778 | 0.933 | 0.143 | 0.74 |
| ***D2S441** | 3 | 5.000 | 4.500 | 0.667 | 0.778 | 0.933 | 0.143 | 0.74 |
| **D10S1248** | 2 | 3.000 | 2.667 | 1.000 | 0.625 | 0.833 | -0.600 | 0.55 |
| ***D13S317** | 3 | 5.000 | 4.500 | 1.000 | 0.778 | 0.933 | -0.286 | 0.74 |
| ***D16S539** | 3 | 4.000 | 3.600 | 0.667 | 0.722 | 0.867 | 0.077 | 0.67 |
| ***D18S51** | 3 | 2.000 | 1.800 | 0.667 | 0.444 | 0.533 | -0.500 | 0.35 |
| ***D2S1338** | 3 | 5.000 | 4.500 | 1.000 | 0.778 | 0.933 | -0.286 | 0.74 |
| ***CSF1PO** | 3 | 2.000 | 1.800 | 0.667 | 0.444 | 0.533 | -0.500 | 0.35 |
| ***Penta D** | 3 | 2.000 | 1.385 | 0.333 | 0.278 | 0.333 | -0.200 | 0.24 |
| ***TH01** | 3 | 3.000 | 2.000 | 0.333 | 0.500 | 0.600 | 0.333 | 0.45 |
| ***vWA** | 3 | 4.000 | 3.000 | 0.667 | 0.667 | 0.800 | 0.000 | 0.62 |
| **D21S11** | 1 | 2.000 | 2.000 | 1.000 | 0.500 | 1.000 | -1.000 | 0.38 |
| ***D7S820** | 3 | 4.000 | 3.000 | 0.667 | 0.667 | 0.800 | 0.000 | 0.62 |
| ***TPOX** | 3 | 2.000 | 1.385 | 0.333 | 0.278 | 0.333 | -0.200 | 0.24 |
| ***D8S1179** | 3 | 3.000 | 2.000 | 0.333 | 0.500 | 0.600 | 0.333 | 0.45 |
| **D12S391** | 1 | 1.000 | 1.000 | 0.000 | 0.000 | 0.000 | #N/A | 0.00 |
| ***D19S433** | 3 | 5.000 | 4.500 | 0.667 | 0.778 | 0.933 | 0.143 | 0.74 |
| ***D22S1045** | 3 | 5.000 | 4.500 | 1.000 | 0.778 | 0.933 | -0.286 | 0.74 |
| ***FGA** | 3 | 4.000 | 3.600 | 1.000 | 0.722 | 0.867 | -0.385 | 0.67 |
| Mean (± SE) | 2.70±0.15 | 3.49±0.29 | 2.95±0.28 | 0.68±0.07 | 0.58±0.05 | 0.76±0.06 | -0.19±0.08 |  |

Na: Observed number of alleles, Ne: effective number of alleles, Ho: observed heterozygosity, He: expected heterozygosity, UHe: unbiased expected heterozygosity, F: fixation index, PIC: Polymorphism information content, * Loci used in establishing genealogical relationship

Table S4. Genealogical relationship of chimpanzees with GlobalFiler™ PCR Amplification Kit, Investigator 24plex QS Kit and PowerPlex® Fusion 6C System

| GlobalFiler™ PCR Amplification Kit | | | | | | | |
| --- | --- | --- | --- | --- | --- | --- | --- |
|  |  |  |  |  | Delta Ln(L) | | |
| **Chimp1** | **Chimp2** | **R** | **LnL(R)** | **U** | **HS** | **FS** | **PO** |
| Buri | Mastan | U | -48.77 | - | 5.72 | 11.35 | 9999 |
| Chhotu | Mastan | U | -48.65 | - | 6.19 | 13.14 | 9999 |
| Chhotu | Buri | U | -48.14 | - | 5.44 | 11.62 | 9999 |
| Investigator 24plex QS Kit | | | | | | | |
|  |  |  |  |  | Delta Ln(L) | | |
| **Chimp1** | **Chimp2** | **R** | **LnL(R)** | **U** | **HS** | **FS** | **PO** |
| Chhotu | Buri | U | -65.94 | - | 7.17 | 15.16 | 9999 |
| Mastan | Buri | U | -67.33 | - | 7.17 | 14.07 | 9999 |
| Mastan | Chhotu | U | -67.8 | - | 8.93 | 18.3 | 9999 |
| PowerPlex® Fusion 6C System | | | | | | | |
|  |  |  |  |  | Delta Ln(L) | | |
| **Chimp1** | **Chimp2** | **R** | **LnL(R)** | **U** | **HS** | **FS** | **PO** |
| Chhotu | Buri | U | -58 | - | 5.65 | 13.04 | 9999 |
| Mastan | Buri | U | -62.21 | - | 8.28 | 17.07 | 9999 |
| Mastan | Chhotu | U | -56.44 | - | 5.77 | 11.39 | 9999 |
